# Therapy of uncomplicated falciparum malaria in Europe: MALTHER – a prospective observational multicentre study

**DOI:** 10.1186/1475-2875-11-212

**Published:** 2012-06-22

**Authors:** Olivier Bouchaud, Nikolai Mühlberger, Philippe Parola, Guido Calleri, Alberto Matteelli, Gabriele Peyerl-Hoffmann, Frédéric Méchaï, Philippe Gautret, Jan Clerinx, Peter G Kremsner, Tomas Jelinek, Annette Kaiser, Anna Beltrame, Matthias L Schmid, Peter Kern, Meike Probst, Alessandro Bartoloni, Thomas Weinke, Martin P Grobusch

**Affiliations:** 1 Infectious and Tropical Diseases Department, Hôpital Avicenne-APHP and Université Paris 13, Bobigny, France; 2 Department of Public Health and Health Technology Assessment, UMIT - University for Health Sciences, Medical Informatics and Technology, Hall i.T, Austria; 3 Infectious and Tropical Medicine Unit, North University Hospital, 13015, Marseille, France; 4 Divisione Malattie Infettive e Tropicali, Ospedale “Amedeo di Savoia”, Torino, Italy; 5 Institute of Infectious and Tropical Diseases, University Hospital, Brescia, Italy; 6 Centre for Infectious Diseases and Travel Medicine, University Hospital Freiburg, Freiburg, Germany; 7 Institute of Tropical Medicine, Antwerp, Belgium; 8 Institute of Tropical Medicine, University of Tübingen, Tübingen, Germany; 9 Berlin Centre for Travel and Tropical Medicine, Berlin, Germany; 10 Institute of Medical Microbiology and Parasitology, University of Bonn, Bonn, Germany; 11 Clinica de Malattie Infettive, AOU di Udine, Udine, Italy; 12 Department of Infection & Tropical Medicine, Royal Victoria Infirmary, Newcastle upon Tyne, UK; 13 Comprehensive Infectious Diseases Center, University Hospitals, Ulm, Germany; 14 Medizinische Klinik m. S. Infektiologie, Charité University Hospital, Berlin, Germany; 15 Infectious and Tropical Diseases Unit, AOU Careggi, and Department of Critical Care Medicine and Surgery, University of Florence, Florence, Italy; 16 Department of Gastroenterology and Infectious Diseases, Klinikum Ernst von Bergmann, Potsdam, Germany; 17 Centre for Tropical and Travel Medicine, Department of Infectious Diseases, Division of Internal Medicine, Amsterdam Medical Centre, University of Amsterdam, Meibergdreef 9, PO Box 22700, 1100 DE, Amsterdam, The Netherlands

**Keywords:** Imported falciparum malaria, Uncomplicated, Therapy, Treatment change, Parasite clearance time, Fever clearance time, Adverse events, Europe

## Abstract

**Background:**

Malaria continues to be amongst the most frequent infectious diseases imported to Europe. Whilst European treatment guidelines are based on data from studies carried out in endemic areas, there is a paucity of original prospective treatment data. The objective was to summarize data on treatments to harmonize and optimize treatment for uncomplicated malaria in Europe.

**Methods:**

A prospective observational multicentre study was conducted, assessing tolerance and efficacy of treatment regimens for imported uncomplicated falciparum malaria in adults amongst European centres of tropical and travel medicine.

**Results:**

Between December 2003 and 2009, 504 patients were included in 16 centres from five European countries. Eighteen treatment regimens were reported, the top three being atovaquone-proguanil, mefloquine, and artemether-lumefantrine. Treatments significantly differed with respect to the occurrence of treatment changes (p = 0.005) and adverse events (p = 0.001), parasite and fever clearance times (p < 0.001), and hospitalization rates (p = 0.0066) and durations (p = 0.001). Four recrudescences and two progressions to severe disease were observed. Compared to other regimens, quinine alone was associated with more frequent switches to second line treatment, more adverse events and longer inpatient stays. Parasite and fever clearance times were shortest with artemether-mefloquine combination treatment. Vomiting was the most frequent cause of treatment change, occurring in 5.5% of all patients but 9% of the atovaquone-proguanil group.

**Conclusions:**

This study highlights the heterogeneity of standards of care within Europe. A consensus discussion at European level is desirable to foster a standardized management of imported falciparum malaria.

## Background

**Figure 1:**
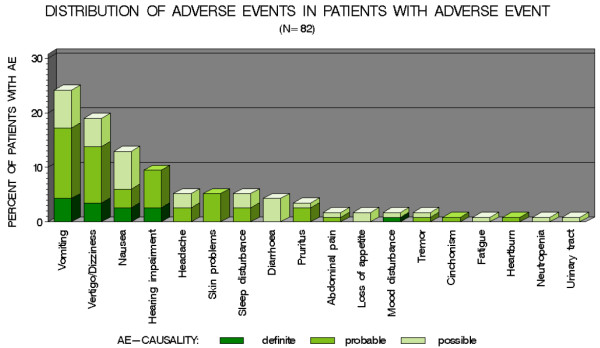
Distribution of adverse events in patients with adverse event

With approximately 11,000 reported cases annually, malaria remains the most important tropical disease imported into Europe both in clinical terms and in absolute figures [1,2]. The majority of cases are caused by *Plasmodium falciparum*, which can lead to complicated disease and death if not treated early. Even in uncomplicated cases, which constitute the majority of cases [3], treatment costs (evaluated in 2005 at €17,416,955/year for 6,321 cases in France) and resulting costs continue to pose a significant strain on the European health systems, which cannot be ignored [4].

Assuming that efficacy and tolerance are similar in imported malaria, the recommended therapy is based on data predominantly derived from therapeutic studies performed in endemic areas [5-14]. Nevertheless, for epidemiological and biological reasons results are not always applicable in the particular field of imported malaria. For example, differences in age and (lack of) background immunity may influence the duration and characteristics (fever and parasite clearance times, adverse events encountered) of the treatment period.

The combination atovaquone-proguanil, largely used in non-endemic countries, has neither been tested extensively in endemic areas nor in non-endemic areas since conducting large studies appears not to be feasible [15-18]. Compared to atovaquone-proguanil and the ‘classical’ drugs, such as quinine, mefloquine or halofantrine, published clinical experience with artemether-lumefantrine for the treatment of uncomplicated falciparum malaria is scarce. Very few studies with limited number of subjects (and only one comparative) are available and, in addition, even if registered throughout Europe in 1999, the drug is still not easily available [19,20]. For example, artemether-lumefantrine was marketed in Germany in 2000, but in France only in 2007, and it is still unavailable in Italy. As a result, use of anti-malarials in non-endemic countries remains, to a certain extent, empirical as comparative studies between competing regimens are lacking.

The overall aim of this multicentre observational study is to summarize data on treatment regimens in Europe, in order to harmonize treatment modalities for uncomplicated falciparum malaria, and to optimize drug treatment strategies amongst European centres of tropical and travel medicine.

## Methods

### Study design and population

The centres contributing to this prospective observational multicentre study were self-selected members of the then clinical surveillance networks TropNetEurop and SIMPID. Patients with uncomplicated falciparum malaria were recruited at the participating centres between December 2003 and December 2009. Criteria for study inclusion were parasitologically confirmed uncomplicated falciparum malaria with or without chemoprophylaxis, no previous malaria therapy for the same episode of illness, and informed consent. Exclusion criteria were age less than 18 years, complicated/severe falciparum malaria on inclusion, or refusal of consent. Pregnancy was not an exclusion criterion due to the observational design of the study. Mixed infections were excluded for comparability reasons.

The primary objective of the study was to describe the profile of anti-malarial regimens used in Europe with a focus on safety and tolerability. Investigated indicators for safety and tolerability were rate and reasons of treatment change, and type, rate and severity of adverse events. Secondary endpoints were clinical and parasitological cure on days 7 and 28, and duration of hospitalization.

Clinical cure was determined as fever clearance time in hours and parasitological cure as parasite clearance time in hours on the grounds of expert microscopy of at least 100 visual fields of a Giemsa-stained thick film.

Allocation to the particular therapeutic regimen was done by the individual participating centres based on country- or centre-specific recommendations, practice pattern, and individual patient’s situation in terms of past exposure, prophylaxis intake, co-morbidity, etc. Randomization was not performed. Doses were given on the basis of manufacturers’ recommendations.

Data were collected anonymously at each participating centre with an electronic entry and reporting tool linked to the regular network surveillance software. In order to assess a standardized and complete minimal dataset from each centre, the tool was equipped with mandatory entry fields, predefined answer categories and data formats and built-in plausibility checks. Treatment outcome was assessed in a standardized follow-up procedure. If feasible, post-discharge control examinations were performed on day 28. If not feasible, a telephone survey was to be carried out on day 28 to detect possible treatment failures and possible late-onset adverse events of drug therapy. The adverse events reporting section of the study software was structured in order to be able to differentiate between signs attributable to the disease itself and to the drug, and to assess their severity. Completed cases were sent electronically to the TropNetEurop/SIMPID coordination centre using the export function of the study software.

### Data analysis

Statistical analysis was performed using SAS software (release 9.1 by SAS Institute Inc., Cary, NC, USA). For each study endpoint the heterogeneity of treatment regimens was first assessed with an overall test. In the case of a significant result, each regimen was tested against all others using a Bonferroni-Holm adjustment for multiple comparisons. Categorical data were analysed using chi-square or Fisher’s exact tests. If asymptotic chi-square tests yielded unreliable results and exact tests were unfeasible due to limited computational resources, Monte Carlo estimates for the exact test based on one million replications were performed. Continuous data were analyzed using a Wilcoxon or Kruskal-Wallis test.

For stratum-adjusted analysis of categorical and continuous data, The Cochran-Mantel-Haenszel test for general association and Friedman’s test were applied, respectively.

### Ethical considerations

An umbrella ethical approval was granted by the site hosting the principal investigator (MPG) at the time point of study initiation. Few centres required, and were granted, additional site-specific approval.

## Results

Five hundred and four malaria patients were included in 16 different centres from five European countries. The contribution of patients by the various centres was heterogeneous, ranging from one to 141. The three main countries providing patients were, by order of number of patients, France, Italy and Germany, respectively.

Patient characteristics are summarized in Additional file 1, stratified by regimen. The majority of patients were migrants living in Europe and visiting friends and relatives (VFR) (69%). Most were infected in West Africa (59.5%). Only 2% contracted *Plasmodium* spp. in endemic areas other than sub-Saharan Africa. Median age was 38 years, with a male:female sex ratio of 1.6. Before travelling, a minority of patients sought medical advice (27%) and/or took an at least partially effective chemoprophylaxis (26%). Median travel duration was one month, with a few patients acquiring malaria with exposure as short as four days. The median delay between onset of symptoms and diagnosis was three days. At time of diagnosis, median temperature was 39.0 °C and parasitaemia varied between very low density to hyperparasitaemia (maximum at 854,000/μL).

A total of 18 different regimens were observed: eight comprising 98% of the prescribed treatments, the 10 other regimens being based either on artemisinine derivatives alone or associated to various anti-malarials in six cases or on quinine associated mainly to pyrimethamine and sulphadoxine at different dosages. Atovaquone-proguanil, artemether-lumefantrine and quinine were used in four countries. Mefloquine was reported only from German and Italian centres, the association β-artemether + mefloquine only in one centre in Italy, intravenous quinine followed by atovaquone-proguanil mainly in one centre in France (in vomiting patients), the combination quinine-clindamycin mainly in another French centre and the combination quinine-tetracycline mainly in Belgium. In 90% of cases, patients received only one regimen.

Overall, most patients were hospitalized (81.2%; n = 409), but differences in the rate of hospitalization between both the different treatment groups (p = 0.0056) and the different countries participating in the study (p < 0.0001) were observed. The rate of hospitalization between countries was as follows: 100% in Belgium (one centre), 98.7% in Germany (eight centres), 87.1% in Italy (four centres), 85.7% in the UK (one centre) and 71.4% in France (three centres).

Globally, the rate of adverse events (at least one event reported) was 16% (n = 82) with differences between treatment groups (p <0.001) (Table 1). Compared to other drugs, quinine alone had a higher rate of adverse events (p = 0.0273). When comparing each drug to each other, quinine administration alone or in association with clindamycin also led to a higher rate of adverse events compared to artemether-lumefantrine (p = 0.0089 and 0.038, respectively). No severe clinical or biological adverse events were reported. The top eight adverse events (each concerning ≥5% of patients) were by numeric decreasing order: vomiting, dizziness, nausea, hearing impairment, headache, skin problems, sleep disturbance and diarrhoea (Figure 1). Overall, 5.5% (n = 28) of the patients experienced vomiting (isolated or in association with other adverse events) with differences between treatment regimens (p = 0.0399). Vomiting was observed with atovaquone-proguanil (n = 20; 71%), mefloquine alone (n = 7; 25%) and in one case with artemether-mefloquine (4%). Hearing impairment and dizziness were associated with quinine. Skin rashes (six cases) were observed with atovaquone-proguanil, mefloquine, quinine alone, quinine + atovaquone-proguanil or clindamycin. Dizziness was reported in 10 cases (10%) of treatment with only mefloquine.

**Table 1:** Tolerability and effectiveness of different treatment regimens in 504 malaria-patients in Europe

	**Total**	AQ + PG^*^	MQ^*^	AR + LU^*^	QUI^*^	AR + MQ^*^	QUI + CL^*^	QUI + TC^*^	QUI + AQPG^*^	Other*	**Overall test** **p-Value**
	**N = 504**	**N = 253**	**N = 97**	**N = 39**	**N = 33**	**N = 20**	**N = 20**	**N = 16**	**N = 15**	**N = 11**	
**Treatment change, N (%)**											0.0053^a^
· no change	451 (89.5)	223 (88.1)	92 (94.9)	37 (94.9)	***25 (75.8)***	19 (95.0)	17 (85.0)	13 (81.3)	15 (100.0)	10 (90.9)	
· due to progress of severity^§^	2 (0.4)	2 (0.8)	0 (0.0)	0 (0.0)	***0 (0.0)***	0 (0.0)	0 (0.0)	0 (0.0)	0 (0.0)	0 (0.0)	
· due to AE other than vomiting	10 (2.0)	2 (0.8)	0 (0.0)	1 (2.6)	***5 (15.2)***	1 (5.0)	1 (5.0)	0 (0.0)	0 (0.0)	0 (0.0)	
· due to vomiting	30 (6.0)	22 (8.7)	5 (5.2)	0 (0.0)	***0 (0.0)***	0 (0.0)	0 (0.0)	3 (18.8)	0 (0.0)	0 (0.0)	
· due to other reasons	11 (2.2)	4 (1.6)	0 (0.0)	1 (2.6)	***3 (9.1)***	0 (0.0)	2 (10.0)	0 (0.0)	0 (0.0)	1 (9.1)	
**Pts. with any AE**^**$**^**, N (%)**	82 (16.3)	34 (13.4)	22 (22.7)	1 (2.6)	**12 (36.4)**	2 (10.0)	7 (35.0)	2 (12.5)	2 (13.3)	0 (0.0)	0.0009 ^a^
**Parasite clearance time**	72	***72***	54	72	48	***36***	48	48	72	48	<0.0001^b^
**Hour median (min-max)**	(0-168)	***(0-168)***	(2-168)	(24-168)	(24-96)	***(12-96)***	(24-96)	(24-108)	(24-168)	(0-96)	
**Fever clearance time,**	48	48	***24***	48	24	***16***	48	***72***	48	36	<0.0001^b^
**Hour median (min-max)**	(0-192)	(0-192)	***(0-168)***	(0-72)	(0-144)	***(0-80)***	(24-96)	***(24-96)***	(0-168)	(4-60)	
**Duration of hospitalization**^**#**^	4	4	4	4	***6***	3.5	***5***	5	3.5	4	0.0002 ^b^
**Day median (min-max)**	(1-18)	(2-15)	(1-18)	(2-7)	***(1-13)***	(1-18)	***(3-7)***	(3-8)	(3-7)	(1-7)	
**Cure rate at day 28**^**&**^	372/376	191/194	73/73	97.0	25/25	9/9	11/11	10/10	13/13	8/8	0.7371^a^
**N cured/N followed-up (%)**	(98.9)	(98.5)	(100.0)	(33)	(100.0)	(100.0)	(100.0)	(100.0)	(100.0)	(100.0)	

a : Monte Carlo estimate for the exact chi-square test (1000000 samples); b : Kruskal-Wallis Test.

Figures in bold italics indicate Bonferroni-Holm adjusted p-values <0.05 in against all other comparison.

* : see additional file 1.

§ : according to the WHO 2000 severity criterion.

$ : includes possible, mild adverse events.

# : N = 401 (95 non hospitalized patients and 8 with missing data).

& : complete case analysis (128 patients lost to follow-up not included in cure rate calculation; numbers of patients with follow-up data are given in the denominators of the cure rate).

Overall, treatment change was necessary in 53 (10%) of the 504 cases; with reasons for change differing between treatment regimens (p = 0.0053). When comparing each regimen to all others, quinine monotherapy was more often associated to change regimen (p 0.0004). Frequency of treatment changes varied from 0 with quinine-atovaquone-proguanil treatment to 24% with quinine monotherapy. Globally, reasons for change were vomiting in 30 cases (57%), adverse events other than vomiting in 10 cases, progression to severe disease in two cases, and various reasons in 11 cases. Vomiting (attributed in 28 cases to adverse events and in two patients treated with atovaquone-proguanil to malaria itself) led to a second-line treatment by intravenous quinine in 24 cases (including nineteen on atovaquone/proguanil and three on mefloquine). Atovaquone-proguanil with a 9% proportion of treatment change due to vomiting differed significantly from the overall average (p = 0.013).

Overall median parasite clearance time (PCT) was 72 hours, with significant differences between treatment groups (p <0.001). When comparing one regimen to the others, the shorter median PCT was observed in patients treated by the association artemether-mefloquine (36 hours; p <0.001) and the longer was with atovaquone-proguanil (72 hours; p < 0.001). In pair wise comparisons adjusted for multiple comparisons, PCT was shorter with artemether-mefloquine compared to atovaquone-proguanil (p < 0.001) and to mefloquine alone (p = 0.026) but not compared to the combination artemether-lumefantrine (p = 0.055).

Median fever clearance time (FCT) was 48 hours with significant differences observed between treatment groups (p <0.001). Compared to all other treatments, median FCT was shorter in patients treated with artemether-mefloquine (p = 0.008) or mefloquine alone (p = 0.001) and was longer in patients treated with quinine-tetracycline (p < 0.001). In multivariate analysis, pair-wise comparisons showed a shorter FCT between artemether-lumefantrine and quinine-tetracycline (p = 0.04), mefloquine and atovaquone-proguanil (p = 0.03), mefloquine and quinine-tetracycline (p < 0.001), artemether-mefloquine, and quinine-tetracycline (p = 0.005).

Duration of hospitalization differed between regimens (p = 0.0002), even after adjusting for country-specific effects (p <0.0001). Median duration was four days, with 25% hospitalized for more than five days, and 5% for more than seven days (maximum of 18 days). Hospitalization was longer with quinine alone (p = 0.0229) or with quinine-clindamycin (p = 0.0156).

The overall cure rate was 74% in the intention-to-treat analysis with a missing data rate due to loss to follow-up of patients of 25%. Excluding those patients lost to follow-up, the cure rate was 99% with four recrudescences occurring in three cases treated with atovaquone-proguanil and in one case treated with artemether-lumefantrine (p > 0.05). Two cases of progression of the disease under treatment were recorded (both with atovaquone-proguanil), with no significant difference between regimens.

## Discussion

This prospective observational study included 504 cases of uncomplicated *P. falciparum* malaria managed in 16 different centres from five countries. This is to date the largest study on imported malaria in terms of numbers of patients. Patient profiles are similar to those observed in the majority of studies on imported malaria [1,2,15-26], except for the fact that there were very few travellers to Asian destinations (e.g. to those areas were mefloquine resistance is emerging). Most of the patients were migrants living in Europe infected in West Africa.

Eighteen different treatment regimens were used. Atovaquone-proguanil was predominantly used (half of cases) followed by older drugs such as mefloquine (19%) or quinine alone or in combination with clindamycin or tetracyclines (14%) [25,27,28]. Artemether-lumefantrine was used in only 8% of cases, but at the beginning of this study, the drug was not easily available in Europe. Taking into account that some European countries do not have national guidelines, the large variety of treatments can be explained by the fact that all participating centres are managed by tropical medicine experienced practitioners who choose therapeutic regimens following different criteria, such as individual risk profiles, personal clinical or research experiences, “local” behaviours, availability of drugs.

A vast majority of patients (81%) were hospitalized, but differences were observed between centres with hospitalization rates from 71% in France to nearly 100% in Belgium or Germany. These differences can be explained by the fact that usages and recommendations differ considerably between countries (Table 2), and with ambulatory treatment criteria remaining controversial [21-24,29,30]. In the present period of restricted health budgets, and in addition to this high rate of hospitalization, the median duration of hospitalization of four days appears relatively long. The longer duration observed with quinine alone or in combination with clindamycin is probably due to the frequency of disabling side effects (dizziness, hearing impairment, headaches) related to a seven-day regimen [31].

**Table 2:** Treatment recommendations and hospitalization policies for imported, uncomplicated falciparum malaria in selected European countries

	**First-line**	**Hospitalization policy**
	**treatment**	**Recommendations (R) or standard practice (SP)**
Belgium	Atovaquone-proguanilArtemether-lumefantrine	SP : hospitalization (ambulatory treatment possible under certain conditions)
	Quinine + cycline	
		
France	Atovaquone-proguanil Artemether-lumefantrine	R : Ambulatory treatment possible on the basis of specific clinical and biological parameters
	
Germany	Atovaquone-proguanil Artemether-lumefantrine	R : hospitalization recommended until treatment completed and patient parasite-free
	Mefloquine	
	
Italy	Atovaquone-proguanil	SP : hospitalization (ambulatory treatment possible under certain conditions)
	Mefloquine	
Spain	Quinine + cycline or clindamycin Atovaquone-proguanil	SP: hospitalization (ambulatory treatment possible under certain conditions)
	
Switzerland	Artemether-lumefantrine Atovaquone-proguanil	SP: hospitalization (ambulatory treatment possible under certain conditions)
	
United Kingdom	Atovaquone-proguanil	R : Systematically, at least 24 h
	Artemether-lumefantrine	
	Quinine + cyclines	
The Netherlands	Atovaquone-proguanil Artemether-lumefantrine	SP: hospitalization (ambulatory treatment possible under certain conditions)
	Quinine + cyclines	
	Chloroquine*	

*selected areas with known chloroquine-sensitive *P. falciparum*, e.g. Central America.

The cure rate of 99% (if patients lost to follow-up were excluded from the analysis) is comparable to that one observed in the literature on imported malaria based on smaller series and equal or higher to studies performed in endemic areas [5-18,32]. However, antimalarial drug resistance (not only against *P. falciparum*, which is in the focus of this paper) continues to emerge on a global scale, already also including artemisinines, thus potentially also jeopardizing the favourable primary treatment outcomes as routinely observed in most travellers.

Four recrudescences were reported in three cases of atovaquone-proguanil treatment and in one case of artemether-lumefantrine treatment; both drugs necessitating to be absorbed with a fatty meal due to the lipophily of, respectively, atovaquone and lumefantrine [33,34]. Details on these relapses are lacking for two cases but for the other two occurring on atovaquone-proguanil, the reason was probably poor absorption due in one case to obesity (115 kg body weight) and consequently under-dosing, and in the second case due to a low atovaquone plasma level in a patient not having taken food with the drug [35]. Consequently, physicians have to stress that with both drugs, their patients should take food.

The rate of patients lost to follow-up at day 28 was high (25%), especially in certain treatment groups where it reached 45% (quinine-clindamycin) or 55% (artemether-mefloquine). Given that recrudescence occurs usually between day 14 and day 30 after treatment, physicians should organize a “recapture” system for these patients, even limited to a call, in order to identify promptly a possible recrudescence [15,19,36].

Under treatment, mean PCT and FCT were 72 and 48 hours, respectively, which is consistent with the literature even if direct comparisons are not easy, since methodology to calculate PCT and FCT may differ between studies [15-20,22,25]. Nevertheless, it appeared that, compared to other regimens including artemether-lumefantrine, both PCT and FCT were shorter in patients treated with artemether-mefloquine, which is not a common regimen for imported malaria (or in endemic areas as well) [13,37,38]. Apart from the bias due to the study design, the difference between both regimens containing artemether can be explained by a higher dosage at day 0 of artemether in the artemether-mefloquine combination compared to artemether-lumefantrine (300 mg and 180 mg, respectively). On the opposite, and not surprisingly so, in view of this combination being slow-acting, PCT and FCT were reported as being longer for atovaquone-proguanil as for other regimens [5,9,15-18]. This is possibly the reason for the reported progression towards severity observed in two atovaquone-proguanil patients, leading to a second-line treatment.

Adverse events were overall relatively frequent (16%), with quinine exhibiting the least favourable tolerance profile. Although none of the adverse events was classified as severe, they constituted the main cause for switching over to second-line treatment in 10% of patients. Vomiting, mainly due to atovaquone-proguanil but also mefloquine, was the leading cause of change and led frequently to an intravenous treatment (quinine).

In the present study, atovaquone-proguanil was by far the most frequent regimen prescribed, providing the opportunity to describe a large cohort of malaria patients treated by this drug in “real-life” conditions. Even if the cure rate was good and statistically comparable to the other regimens, three relapses and two cases of progression to severe malaria were observed, possibly due to poor absorption. In addition, PCT was longer compared to the other regimens. The rate of adverse events was 13%, better than that one of quinine but vomiting appeared to be specifically related to that regimen leading in a high proportion to a second-line treatment. Surprisingly, these digestive side-effects were not clearly reported in studies both performed in endemic areas or in non-endemic areas and were only suggested in one study on imported malaria [5,9,15,17,18,32,39,40].

Whilst this study constitutes the largest cohort reported on falciparum malaria imported to Europe to date, it has numerous limitations mainly due to its observational design and its overall limited number of observations; and the patient collective being heterogeneous with “centre effects”. For example, it was impossible to standardize timing of follow-up testing for fever and parasite clearance. Consequently, comparisons between treatment groups or countries, even statistically significant, have to be carefully interpreted. Nevertheless, the methodology used here is the only way to observe a large cohort of imported-malaria patients in routine conditions as data in this specific field of malaria are very limited. To that end, the collection of observational data on the scale of what is presented here should be perpetuated and extended to additional sites involved in the treatment of malaria imported into Europe.

## Conclusions

Despite limitations in its design, this observational multicentre European study of 504 patients with uncomplicated falciparum malaria offers relevant data on management of imported malaria in Europe. The hospitalization rate appeared high with heterogeneity between centres. The number of treatment regimens was as high as eighteen. Atovaquone-proguanil, the most prescribed drug in this study, appeared to be a valuable option but poor absorption, delay in PCT and FCT and vomiting, frequently inducing a second-line treatment are limiting factors. Due to a poor tolerance profile possibly leading to prolongation of hospitalization, quinine alone should not be recommended any more as a first-line treatment. The study results, by highlighting the current diversity of actual treatments against a backdrop of national and supranational guidelines which favour few established regimens, should stimulate and an intensified consensus discussion at European level, which is desirable in order to more strictly select a limited number of treatments and to foster standardized management of imported malaria.

## Competing interests

The authors declare they have no conflict of interest.

## Authors’ contributions

OB contributed to the design of the study, contributed patient data and led writing the paper. NM designed the database, served as the study data manager, analysed the data and contributed to writing of the first draft and final version of the paper. AM contributed to the design of the study, to patient data collection and to the writing of the final paper. All other authors contributed patient data and contributed to the writing of the final paper. MPG conceived and designed the study, served as Principal Investigator and contributed to the writing of the first draft and final version of the paper. All authors read and approved the final manuscript.

## Supplementary Material

Additional file 1 Main characteristics of 504 malaria patients in different European settings according to their treatment regimen. Click here for file
