# Supplementary material for: Therapy of uncomplicated falciparum malaria in Europe: MALTHER – a prospective observational multicentre study
Source: Malar J. 2012 Jun 22;11:212. doi: 10.1186/1475-2875-11-212 (PMC3477029; doi:10.1186/1475-2875-11-212)
Supplement: Additional file 1 — Main characteristics of 504 malaria patients in different European settings according to their treatment regimen. [file 1475-2875-11-212-S1.pdf]

Table 1: Main characteristics of 504 malaria patients in different European settings according to their treatment regimen

	Total<br><br>N=504<br>(100%)	AQ+PG[1]<br><br>N=253<br>(50%)	MQ[2]<br><br>N=97<br>(19%)	AR+LU[3]<br><br>N=39<br>(8%)	QUI[4]<br><br>N=33<br>(6.5%)	AR+MQ[5]<br><br>N=20<br>(4%)	QUI+CL[6]<br><br>N=20<br>(4%)	QUI+TC[7]<br><br>N=16<br>(3%)	QUI+AQPG[8]<br><br>N= 15<br>(3%)	Other[9]<br><br>N=11<br>(2%)
Sex ratio*<br>(M/F)	1.6	2	2	1.1	0.4	1.2	1.5	1.6	2	1.2
Age median<br>(range)	38<br>(18-79)	39<br>(18-71)	37<br>(18-72)	39<br>(20-69)	37<br>(18-69)	41<br>(25-79)	31<br>(19-65)	39<br>(18-69)	39<br>(19-67)	36<br>(20-62)
Inpatient° %*<br>(N)	81.2<br>(409)	75.9<br>(192)	84.5<br>(82)	74.4<br>(29)	90.9<br>(30)	100<br>(20)	100<br>(20)	100<br>(16)	90.9<br>(30)	72.7<br>(8)
Co-morbidity°%*<br>(N)	73.4<br>(370)	75.5<br>(191)	73.2<br>(71)	69.2<br>(27)	78.8<br>(26)	60<br>(12)	85<br>(17)	37.5<br>(6)	73.3<br>(11)	81.8<br>(9)
Pre-travel advice° %*<br>(N)	27.4<br>(138)	32.8<br>(83)	13.4<br>(13)	33.3<br>(13)	45.5<br>(15)	5<br>(1)	50<br>(10)	0<br>(0)	13.3<br>(2)	9.1<br>(1)
Chemoprophylaxis°%*<br>(N)	26.2<br>(132)	34.8<br>(88)	10.3<br>(10)	30.8<br>(12)	27.3<br>(9)	5<br>(1)	45<br>(9)	0<br>(0)	13.3<br>(2)	9.1<br>(1)
Travel purpose %*(N)										
• Tourism	10.9(55)	9.1(23)	9.3(9)	2O.5(8)	12.1(4)	25(5)	10(2)	18.8(3)	0(0)	9.1(1)
• VFR**	69(348)	75.9(192)	68(66)	53.8(21)	66.7(22)	55(11)	60(12)	43.8(7)	66.7(10)	63.6(7)
• Business	7.1(36)	7.1(18)	5.2(5)	7.7(3)	6.1(2)	5(1)	10(2)	18.8(3)	13.3(2)	0(0)
• Humanitarian	5.8(29)	3.6(9)	6.2(6)	10.3(4)	6.1(2)	10(2)	15(3)	0(0)	13.3(2)	9.1(1)
• Other***	7(36)	4(11)	11(11)	7(3)	9(3)	5(1)	5(1)	18(3)	6(1)	18(2)
Region of infection°§<br>%*(N)										
• West Africa	59.5(300)	52.6(133)	80.4(78)	59(23)	36.4(12)	85(17)	40(8)	81.3(13)	53.3(8)	72.7(8)
• Central Africa	13.5(68)	15(38)	11.3(11)	20.5(8)	3(1)	10(2)	10(2)	12.5(2)	20(3)	9.1(1)
• East Africa	18(3.6)	2(5)	6.2(6)	7.7(3)	6.1(2)	5(1)	0(0)	0(0)	0(0)	9.1(1)
• South Africa	11(2.2)	0.8(2)	1(1)	7.7(3)	3(1)	0(0)	0(0)	6.3(1)	13;3(2)	9.1(1)
• Madagascar and	19(96)	26.5(67)	0(0)	5.1(2)	48.5(16)	0(0)	50(10)	0(0)	6.7(1)	0(0)

Figure 2

Comoro<br>• Caribbean<br>• Indian sub-continent<br>• South East Asia	0.6(3)<br><br>0.6(3)<br>0.8(4)	0.4(1)<br><br>1.2(3)<br>1.6(4)	0(0)<br><br>0(0)<br>0(0)	0(0)<br><br>0(0)<br>0(0)	3(1)<br><br>0(0)<br>0(0)	0(0)<br><br>0(0)<br>0(0)	0(0)<br><br>0(0)<br>0(0)	0(0)<br><br>0(0)<br>0(0)	6.7(1)<br><br>0(0)<br>0(0)	0(0)<br><br>0(0)<br>0(0)
Travel duration, day median (min/max)	30 (4/>365)	30 (4/>365)	28 (7/>365)	29 (9/300)	45 (10/>365)	30 (15/335)	45 (9/130)	30 (14/365)	25 (7/300)	28 (7/360)
Delay from symptoms to diagnosis°, day median (min/max)	3 (0/92)	3 (0/38)	3 (0/92)	2 (0/15)	5 (1/65)	3 (0/66)	5 (1/26)	5 (2/19)	4 (0/12)	3 (0/35)
Temperature at diagnosis, °C median (min/max)	39 (36/42)	39 (36/41)	39 (36/41)	39 (36/41)	39 (36/41)	39 (36/41)	39 (38/40)	38 (36/40)	39 (36/40)	39 (37/40)
Parasite density at diagnosis°, per µL median (min#/max)	10,000 (0 /854,000)	6,292 (48 /364,000)	9,176 (1 /231,000)	18,500 (48 /364,000)	9,000 (400 /200,000)	14,010 (968 /197,000)	22,750 (1985 /854,000)	40,000 (240 /218,400)	16,000 (50 /245,000)	22,000 (61 /71,100)
Country of treatment° %*(N)<br>• Belgium<br>• France<br>• Germany<br>• Italy<br>• United Kingdom	 <br>5.2(26)<br>53.4(269)<br>15.5(78)<br>24.6(124)<br>1.4(7)	 <br>2(5)<br>76.3(193)<br>11.9(30)<br>9.9(25)<br>0(0)	 <br>0(0)<br>1(1)<br>30.9(30)<br>68(66)<br>0(0)	 <br>0(0)<br>51.3(20)<br>38.5(15)<br>7.7(3)<br>2.6(1)	 <br>6.1(2)<br>75.8(25)<br>3(1)<br>9.1(3)<br>6.1(2)	 <br>0(0)<br>0(0)<br>11.9(30)<br>100(20)<br>0(0)	 <br>5(1)<br>95(19)<br>0(0)<br>0(0)<br>0(0)	 <br>87.5(14)<br>0(0)<br>0(0)<br>12.5(2)<br>0(0)	 <br>26.7(4)<br>73.3(11)<br>0(0)<br>0(0)<br>0(0)	 <br>0(0)<br>0(0)<br>18.2(2)<br>45.5(5)<br>36.4(4)

1. AQ+PG: atovaquone+proguanil (standard dosage according to the manufacturer); 2. MQ: mefloquine (standard dosage according to the manufacturer); 3. AR+LU: artemether+lumefantrine (standard dosage according to the manufacturer); 4. QUI: IV or oral quinine (24 mg/kg/d /7 days); 5. AR+MQ: β-artemether (300 mg loading dose at D0 + 100 mg/day until parasite-negative) + mefloquine (standard dosage); 6. QUI+CL: quinine (8 mg/kg/8 h for 3 days) + clindamycin (10 mg/kg/8h for 3 days); 7. QUI+TC: quinine (600 mg/8 h/d for 5-7 days) + tetracyclines (200 mg/d for 7 days); 8. QUI+AQPG: IV quinine (24 mg/kg/d for 1 or 2 days followed by 1 course of atovaquone+proguanil at standard dosage; used in case of vomiting due to malaria); 9. other: see text

°: significant heterogeneity among treatment groups at 5% alpha-level

*: percentages are calculated relatively to each treatment group

**: migrants visiting friends and relatives
***: including recent immigration, students, soldiers
§: one case reported as coming from North Africa not included in the table
#: thick film negative but with a positive dipstick or a positive Plasmodium falciparum serology with a typical history of malaria in a non-immune patient
